# Zinc protoporphyrin IX, a heme oxygenase-1 inhibitor, demonstrates potent antitumor effects but is unable to potentiate antitumor effects of chemotherapeutics in mice

**DOI:** 10.1186/1471-2407-8-197

**Published:** 2008-07-11

**Authors:** Dominika Nowis, Marek Bugajski, Magdalena Winiarska, Jacek Bil, Angelika Szokalska, Pawel Salwa, Tadeusz Issat, Halina Was, Alicja Jozkowicz, Jozef Dulak, Tomasz Stoklosa, Jakub Golab

**Affiliations:** 1 Department of Immunology, Center of Biostructure Research, the Medical University of Warsaw, Banacha 1A, 02-097 Warsaw, Poland; 2 Department of Laboratory Diagnostics and Clinical Immunology, the Medical University of Warsaw, Marszalkowska 24, 00-576 Warsaw, Poland; 3 Department of Medical Biotechnology, Faculty of Biochemistry, Biophysics and Biotechnology, Jagiellonian University, Gronostajowa 7, 30-387 Krakow, Poland

## Abstract

**Background:**

HO-1 participates in the degradation of heme. Its products can exert unique cytoprotective effects. Numerous tumors express high levels of HO-1 indicating that this enzyme might be a potential therapeutic target. In this study we decided to evaluate potential cytostatic/cytotoxic effects of zinc protoporphyrin IX (Zn(II)PPIX), a selective HO-1 inhibitor and to evaluate its antitumor activity in combination with chemotherapeutics.

**Methods:**

Cytostatic/cytotoxic effects of Zn(II)PPIX were evaluated with crystal violet staining and clonogenic assay. Western blotting was used for the evaluation of protein expression. Flow cytometry was used to evaluate the influence of Zn(II)PPIX on the induction of apoptosis and generation of reactive oxygen species. Knock-down of HO-1 expression was achieved with siRNA. Antitumor effects of Zn(II)PPIX alone or in combination with chemotherapeutics were measured in transplantation tumor models.

**Results:**

Zn(II)PPIX induced significant accumulation of reactive oxygen species in tumor cells. This effect was partly reversed by administration of exogenous bilirubin. Moreover, Zn(II)PPIX exerted potent cytostatic/cytotoxic effects against human and murine tumor cell lines. Despite a significant time and dose-dependent decrease in cyclin D expression in Zn(II)PPIX-treated cells no accumulation of tumor cells in G1 phase of the cell cycle was observed. However, incubation of C-26 cells with Zn(II)PPIX increased the percentage of cells in sub-G1 phase of the cells cycle. Flow cytometry studies with propidium iodide and annexin V staining as well as detection of cleaved caspase 3 by Western blotting revealed that Zn(II)PPIX can induce apoptosis of tumor cells. B16F10 melanoma cells overexpressing HO-1 and transplanted into syngeneic mice were resistant to either Zn(II)PPIX or antitumor effects of cisplatin. Zn(II)PPIX was unable to potentiate antitumor effects of 5-fluorouracil, cisplatin or doxorubicin in three different tumor models, but significantly potentiated toxicity of 5-FU and cisplatin.

**Conclusion:**

Inhibition of HO-1 exerts antitumor effects but should not be used to potentiate antitumor effects of cancer chemotherapeutics unless procedures of selective tumor targeting of HO-1 inhibitors are developed.

## Background

Heme oxygenase (HO) is a microsomal enzyme that catalyzes oxidative cleavage of the porphyrin ring in heme molecule leading to the formation of biliverdin, carbon monoxide (CO) and free iron [1,2]. Biliverdin is further converted into bilirubin by biliverdin reductase. All HO products exert pleiotropic effects including numerous cytoprotective responses [3]. Bilirubin and biliverdin are among the most potent endogenous scavengers of reactive oxygen species (ROS) [4]. CO exerts strong antiapoptotic and anti-inflammatory effects through induction of soluble guanylyl cyclase. It suppresses production of tumor necrosis factor (TNF), interleukin-1β (IL-1β) and CCL4 chemokine (macrophage inflammatory protein-1β), but up-regulates synthesis of anti-inflammatory IL-10 [5]. Finally, free iron (Fe^2+^) despite participation in Fenton reaction that leads to formation of highly reactive hydroxyl radicals, also activates Fe-ATPase, a transporter that removes intracellular iron, as well as induces expression of ferritin heavy chains which sequester free iron and exert specific cytoprotective roles [6].

Two isoforms of heme oxygenase exist. HO-1 is an inducible enzyme that belongs to the heat shock protein (HSP32) family. Its expression is induced by a vast array of stress-inducing stimuli that include: oxidative stress, heat shock, UV irradiation, exposure to heavy metals and numerous other toxins, including chemotherapeutics [7]. Some observations indicate that HO-1 and its products also exert anti-inflammatory effects and participate in the control of growth and proliferation of tumor cells. Elevated constitutive levels of HO-1 have been observed in a number of human tumors including glioma, melanoma, prostate, pancreatic and renal cell carcinoma, lymphosarcomas, Kaposi sarcoma and hepatoma [7]. Enhanced expression of HO-1 can also contribute to tumor progression through promotion of angiogenesis and metastases formation [8,9]. Furthermore, the increased basal level of HO-1 expression in tumor cells can be further elevated by chemotherapeutics, radiotherapy or photodynamic therapy [10,11].

Altogether HO products participate in attenuation of oxidative stress, suppression of inflammatory responses, inhibition of apoptosis and promotion of angiogenesis [12,13]. Therefore, accumulating evidence indicates that HO-1 can be a therapeutic target for antitumor treatment. Indeed, it was shown that zinc protoporphyrin IX (Zn(II)PPIX) or its pegylated derivative, a potent HO inhibitor, can exert significant antitumor effects against several tumors in mice [14-16]. Moreover, inhibition of HO-1 expression or activity was shown to increase responsiveness of tumor cells to other anticancer treatments *in vitro *[10,16,17]. The aim of these studies was to explore the *in vivo *role of HO-1 in tumor growth and in protecting tumor cells against chemotherapeutics.

## Methods

### Tumor cells

Human ovarian carcinoma (MDAH2774), human pancreatic adenocarcinoma (Mia PaCa2), human breast carcinoma (MDA-MB231), murine breast carcinoma (EMT6) cell lines were purchased from ATCC (Manassas, VA, USA). Murine colon-26 (C-26), a poorly differentiated colon adenocarcinoma cell line was obtained from prof. Danuta Dus (Institute of Immunology and Experimental Medicine, Wroclaw, Poland). B16F10 murine melanoma cells were provided by Dr. M. Kubin (Wistar Institute, Philadelphia, PA). Cells were cultured in RPMI 1640 medium (C-26 and B16F10) (Invitrogen, Carlsbad, CA, USA) or DMEM (MDAH2774, Mia PaCa2, MDA-MB231 and EMT6) supplemented with 10% heat-inactivated fetal calf serum, antibiotics, 2-mercaptoethanol (50 μM) and L-glutamine (2 mM) (all from Invitrogen), hereafter referred to as culture medium.

### Reagents

Zinc (II) propoporphyrin IX (Zn(II)PPIX), a HO-1 inhibitor, was purchased from Frontier Scientific Europe Ltd. (Carnforth, Lancashire, United Kingdom) and was dissolved in dimethylsulfoxide (DMSO) (Sigma, St. Louis, USA) to the final stock concentration of 5 mM. Bilirubin (Sigma, St Louis, MO, USA) was dissolved in 0.1 N NaOH to the final stock concentration of 10 mM. Hemin (from Sigma) was dissolved in 0.1 N KOH to the final stock concentration of 10 mM. All the solutions were prepared in the dark right before adding to the cell cultures.

### Mice

Female BALB/c and C57Bl/6 mice, 8–12 weeks of age were used for *in vivo *experiments. Breeding pairs were obtained from the Institute of Oncology (Warsaw, Poland). Mice were kept in conventional conditions with full access to food and water during experiments. All of the animal studies were performed in accordance with the guidelines approved by the Ethical Committee of the Medical University of Warsaw.

### Western blotting

For Western blotting C-26 were treated with Zn(II)PPIX for 24, 48 or 72 h. After indicated times of culture cells were washed with PBS and lysed with radioimmunoprecipitation assay buffer (RIPA) containing Tris base 50 mM, NaCl 150 mM, NP-40 1%, sodium deoxycholate 0.25% and EDTA 1 mM supplemented with Complete^® ^protease inhibitor cocktail tablets (Roche Diagnostics, Mannheim, Germany). Protein concentration was measured using Bio-Rad Protein Assay (BioRad, Hercules, CA, USA). Equal amounts of whole cell proteins were separated on 12% SDS-polyacrylamide gel, transferred onto Protran^® ^nitrocellulose membranes (Schleicher and Schuell BioScience Inc., Keene, NH, USA), blocked with TBST [Tris buffered saline (pH 7.4) and 0.05% Tween 20] supplemented with 5% nonfat milk and 5% FBS. The following antibodies at 1:1000 dilution were used for the 24 h incubation: mouse monoclonal anti-α-tubulin (Promega Corporation, Madison, WI, USA), rabbit polyclonal anti-cleaved caspase 3 (Cell Signaling Technology, Beverly, MA, USA) and mouse monoclonal anti-cyclin D1 (Santa Cruz). After extensive washing with TBST, the membranes were incubated for 45 min with corresponding alkaline phosphatase-coupled secondary antibodies (from Jackson Immuno Research). The color reaction for alkaline phosphatase was developed using nitroblue tetrazolium and 5-bromo-4-chloro-3-indolyl phosphate (Sigma).

### Cytostatic/cytotoxic assay

The cytostatic and/or cytotoxic effects were measured using crystal violet staining as described [18]. Briefly, tumor cells were dispensed into 96-well plates (Sarstedt, Numbrecht, Germany) at a concentration of 5 × 10^3 ^cells per well and allowed to attach overnight. The following day investigated agents were added at indicated concentrations. Cells were kept in dark for 48 or 72 h. After the incubation time the cells were rinsed with PBS and stained with 0.5% crystal violet in 2% ethanol for 10 min at room temperature. Plates were washed four times with tap water and the cells were lysed with 1% SDS solution. Absorbance was measured at 595 nm using an enzyme-linked immunosorbent assay reader (SLT Labinstrument GmbH, Salzburg, Austria), equipped with a 595 nm filter. Cytotoxicity was expressed as relative viability of tumor cells (% of control cultures incubated with medium only) and was calculated as follows: relative viability = (A_e _- A_b_) × 100/(A_c _- A_b_), where A_b _is the background absorbance, A_e _is experimental absorbance, and A_c _is the absorbance of untreated controls.

### Clonogenic assay

MDAH2774 or Mia PaCa2 cells were plated at 2.5 × 10^5 ^cells per 35-mm dish (Sarstedt). Four hours after seeding, Zn(II)PPIX was added at indicated concentrations. After 24 hours of incubation in dark, the cells were washed with PBS, trypsinized and seeded into 35-well plates in triplets at the concentration of 1 × 10^3 ^cells per a dish. Fresh medium containing Zn(II)PPIX was added. The medium was removed daily for the 6 following days. After 14 day incubation in the dark, the cells were rinsed with PBS, fixed for 10 min in pure methanol and stained with 0.5% crystal violet in 2% ethanol for 10 min at room temperature. Then the plates were washed four times with tap water and air-dried. The images of the plates were made using the Olympus Camedia C750 Ultra Zoom digital camera.

### FACS analysis of cell cycle and apoptosis

For the cell cycle analysis, C-26 cells were seeded into 6-well plates (Sarstedt) at the concentrations 1 to 3 × 10^5 ^cells per well and incubated in dark with 2.5 μM or 5 μM Zn(II)PPIX. The incubation time varied from 24 to 72 h. After the incubation, the cells were washed with PBS, trypsinized and centrifuged at 1000 rpm for 10 min in 4°C. The pellet was resuspended in 0.5 ml of PBS and injected under the surface of ice cold 70% ethanol for 24-h fixation in -20°C. At the day of analysis, the cells were washed from ethanol, stained with 5 μg/ml propidium iodide (PI, Becton Dickinson, Mountain View, CA, USA) at the presence of RNase A (Becton Dickinson) for 30 min at 37°C. For the analysis of apoptosis induction C-26 or Mia PaCa2 cells were seeded into 6-well plates (Sarstedt) at the concentrations 1 to 3 × 10^5 ^cells per well and incubated in dark with 2.5 μM or 5 μM Zn(II)PPIX. The incubation time varied from 6 to 72 h. After the incubation, the cells were washed with PBS, trypsinized and centrifuged at 1000 rpm for 10 min in 4°C. The pellet was resuspended in 0.5 ml of Annexin-binding buffer (BioSource International, Camarillo, CA, USA) and then incubated for 15 min with 5 μl of Annexin V-FITC (BioSource). Finally, the cells were stained with 5 μg/ml PI. The cytofluorometric analysis was performed using FACSCalibur (Becton Dickinson). For single analysis 1 × 10^4 ^cells were used. Data were collected at the wavelength of 580 nm (for PI) and 520 nm for FITC, and analysed with CELLQuest 1.2 software (Becton Dickinson).

### HO-1 RNAi

Small interfering RNA against murine HO-1 was obtained by chemical synthesis from Dharmacon (Lafayette, CO, USA). The following sequence was used for targeting of murine HO-1: 5'-GCA-GAA-CCC-AGU-CUA-UGC-C-3'. For RNAi experiments 3,5 × 10^5 ^C-26 cells were seeded into 6-well plates (Sarstedt). After 24 h cells were washed with Optimem™ medium (Invitrogen) and transfected with 100 nM siRNA using OligofectAMINE™ according to the manufacturer's protocol. 10 μM hemin was added to the appropriate groups 2 h befor RNAi procedure. After 8 h trasfection medium was replaced with full DMEM culture medium. 24 h after transfection cells were harvested and ROS generation analysis was performed (see below).

### ROS generation assay

For determination of ROS generation, C-26 cells (3,5 × 10^5 ^cells per well) were seeded into 6-well paltes (Sarstedt) and treated for 24 h with 2.5 μM Zn(II)PPIX and/or 50 μM biliubin or subjected to HO-1 RNAi (as described above). On the day of the analysis, cells were trypsynized, washed 3 times in ice-cold PBS and resuspended in 1 ml of PBS. One μl of CM-H_2_DCFDA (Invitrogen, 5 mM DMSO solution) was added to each sample for 20 min incubation at 37°C. Then, cells were washed twice with ice-cold PBS and subjected to flow cytometry using FACSCalibur (Becton Dickinson). For single analysis 1 × 10^4 ^cells were used. Data were collected at the wavelength of 520 nm and analysed with CELLQuest 1.2 software (Becton Dickinson).

### Tumor treatment and monitoring

For assessment of antitumor activity of Zn(II)PPIX *in vivo*, exponentially growing C-26 were harvested, re-suspended in PBS medium to the appropriate concentration, and injected at the dose of 1 × 10^5 ^cells per mouse into the footpad of the right hind limb of experimental mice. Tumor cell viability measured by trypan blue exclusion was always above 95%. For *in vivo *treatment Zn(II)PPIX was dissolved in DMSO and further diluted in 0.9% NaCl to required concentrations. Final DMSO concentration was always less then 0.1%. Zn(II)PPIX was distributed intraperitoneally at doses from 12.5 to 50 mg per kg of body weight or orally at doses from 11 to 22 mg per kg of body weight. Control animals received 0.1% DMSO solution in 0.9% NaCl i.p. or orally.

For *in vivo *experiments evaluating the effectiveness of combine treatment using Zn(II)PPIX and chemotherapeutics, exponentially growing C-26, EMT6 and B16F10 cells were injected at the dose of 1 × 10^5^, 1 × 10^5 ^and 1 × 10^6 ^cells per mouse, respectively into the footpad of the right hind limb of experimental mice. Zn(II)PPIX treatment (50 mg/kg i.p.) was started on the day 7 after inoculation of tumor cells and continued for 7 consecutive days. First dose of HO-1 inhibitor was administered 1 h before each of the chemotherapeutics to eliminate any possible interactions (such as neutralization) between drugs. Cisplatin (Platidam, Pliva-Lachema, Cech Republic) at the dose of 7.5 mg/kg i.p., 5-FU (Fluorouracil 1000 Medac, Medac, Hamburg, Germany) – 50 mg/kg i.p., or doxorubicin (Adriblastina RD, Pharmacia Italia, Milan, Italy) – 7.5 mg/kg i.p. were administered at a single dose on the day 7^th ^after inoculation of tumor cells.

For *in vivo *experiments determining influence of HO-1 gene transfer on sensitivity of B16F10 melanoma cells to cisplatin treatment, exponentially growing B1 (HO-1 transfected) and B5E (empty plasmid transfected) cells were injected at the dose of 1 × 10^6 ^cells per mouse into the footpad of the right hind limb of experimental mice. Cisplatin (Platidam, Pliva-Lachema) was administered i.p. at a single dose (2.5, 5.0 or 7.5 mg/kg) on the day 7^th ^after inoculation of tumor cells.

For *in vivo *experiments exponentially growing B1 and B5E cells were injected at a dose of 1 × 10^6 ^cells per mouse into the footpad of the right hind limb of experimental mice. Cisplatin was administered i.p. at a single dose of 7.5 mg/kg on the 7^th ^day after inoculation of tumor cells. Zn(II)PPIX treatment (50 mg/kg i.p.) was started on the day 7 after inoculation of tumor cells and continued for 7 consecutive days. First dose of HO-1 inhibitor was administered 1 h before the chemotherapeutic.

In all the experiments mentioned above, local tumor growth was determined every second day as described previously [19] by the formula: tumor volume (mm^3^) = (longer diameter) × (shorter diameter)^2^.

### Statistical analysis

Data were calculated using Microsoft™ Excel 2003. Differences in *in vitro *cytotoxicity assays and tumor volume were analyzed for significance by Student's *t *test. Significance was defined as a two-sided *P *< 0.03.

## Results

### Zn(II)PPIX induces potent cytostatic/cytotoxic effects against murine and human tumor cells

**Figure 1:**
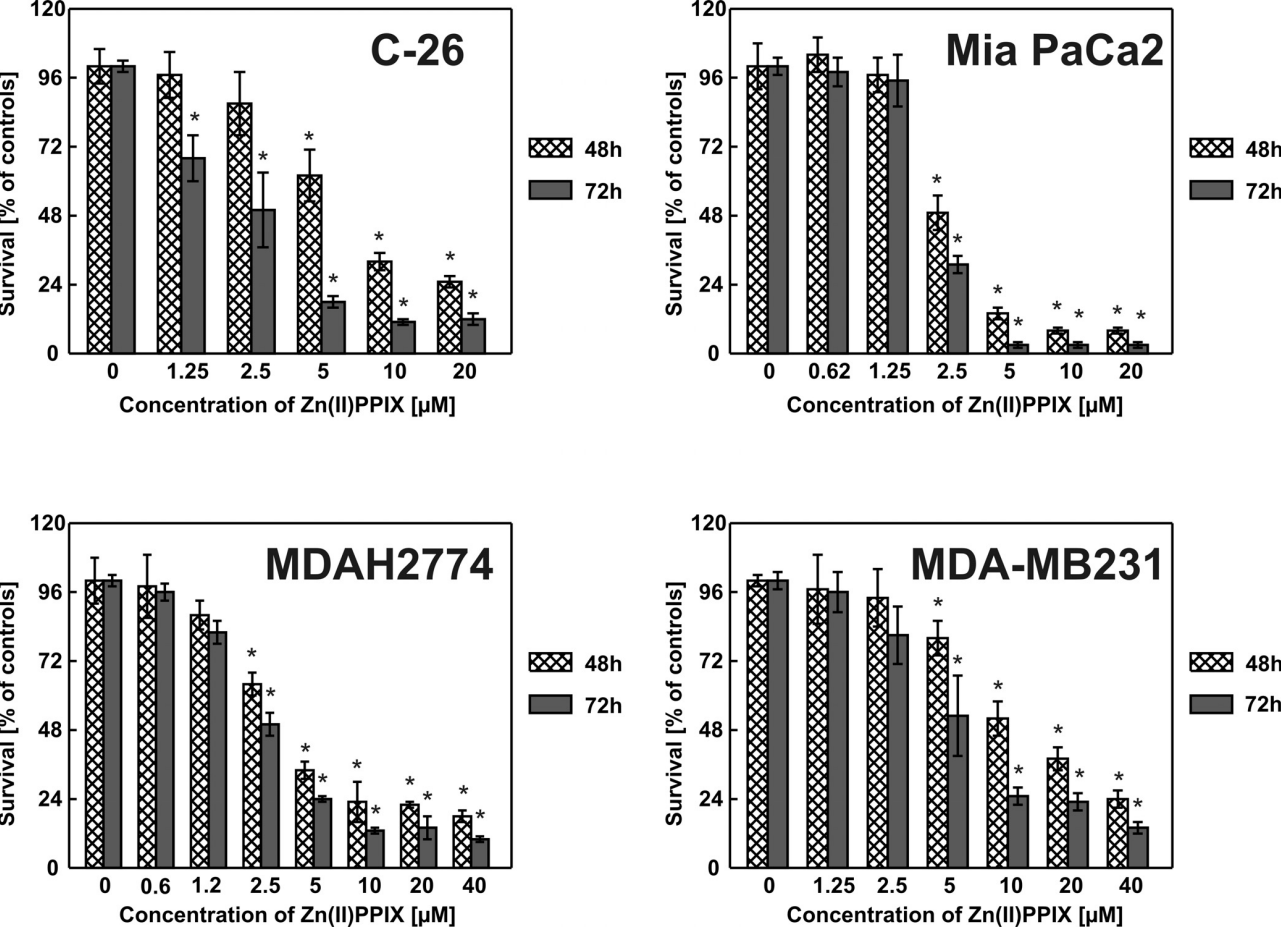
**Zn(II)PPIX exerts cystostatic/cytotoxic effects against tumor cells**. Tumor cells were incubated with serial dilutions of Zn(II)PPIX for 48 or 72 hours. The cytostatic and/or cytotoxic effects of treatment were measured with a standard crystal violet staining assay. Bars represent means ± SD. *P < 0.05 (two way Student's *t*-test) in comparison with controls.

**Figure 2:**
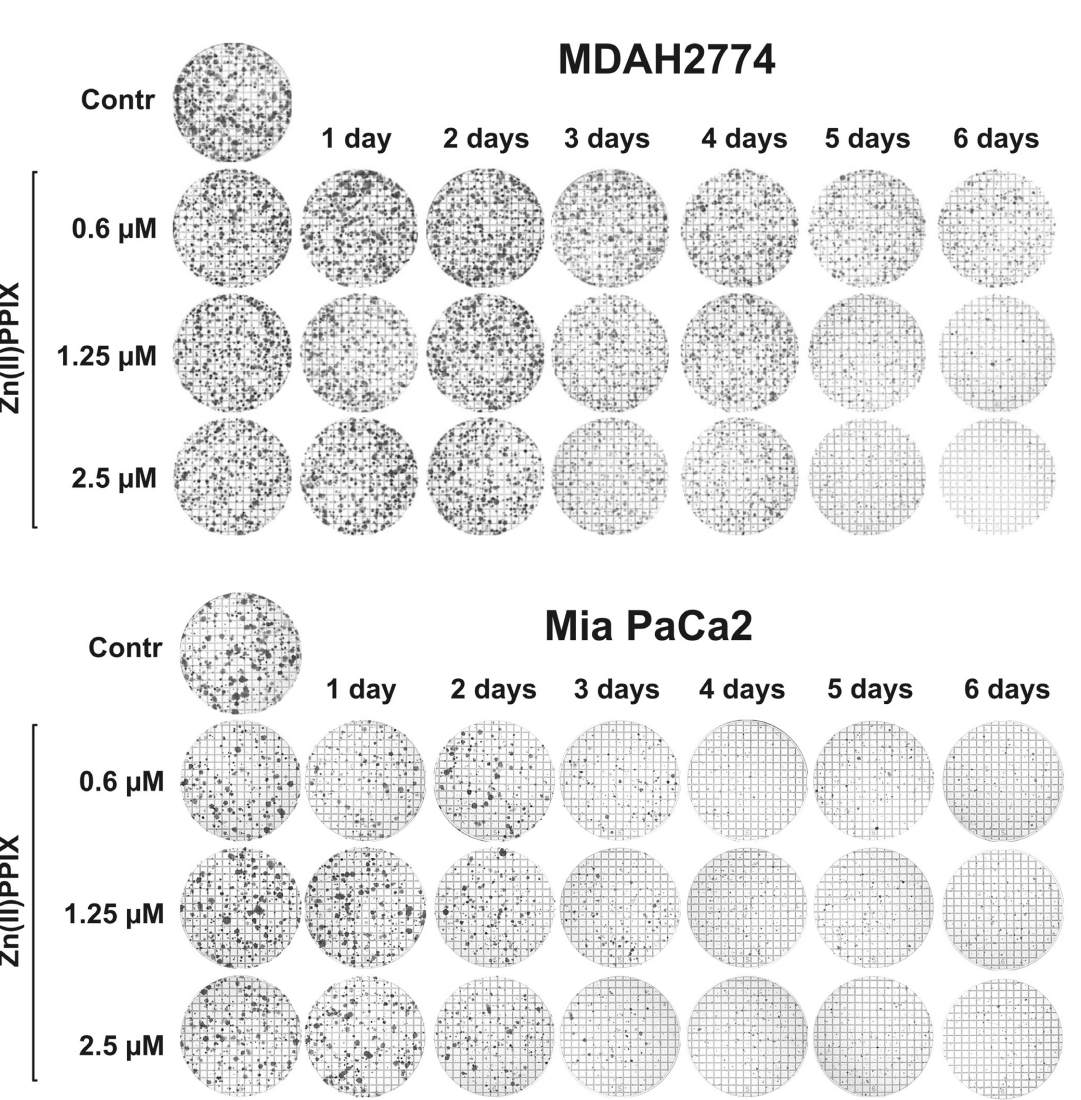
**The influence of Zn(II)PPIX on formation of tumor cell colonies**. For the clonogenic assay, MDAH2774 or Mia PaCa2 cells were plated at a concentration of 1 × 10^3 ^cells/dish. Medium containing Zn(II)PPIX was replaced daily for 1–6 consecutive days. On day 14 after PDT, the plates were fixed with methanol and stained with crystal violet.

Four different cell lines of murine (C-26, colon adenocarcinoma) and human (Mia PaCa2, a pancreatic cancer, MDAH2774, ovarian carcinoma, and MDA-MB231, breast carcinoma) origin were incubated with increasing concentrations of Zn(II)PPIX for 48 and/or 72 hours. HO-1 inhibitor exerted dose- and time-dependent cytostatic/cytotoxic effects as measured with crystal violet staining (Fig. 1). Similar effects were observed in MTT assay (not shown) and in a clonogenic assay performed with Mia PaCa2 and MDAH2774 cells (Fig. 2).

**Figure 3:**
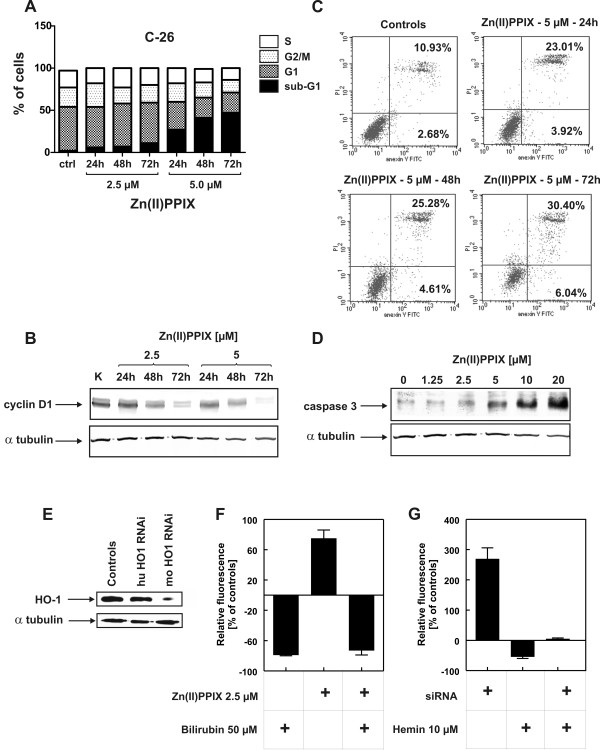
**Zn(II)PPIX induces apoptosis and increases formation of reactive oxygen species**. C-26 cells were grown in Petri dishes for 24 hours before addition of Zn(II)PPIX for the indicated time. [A] Cell cycle analysis was performed by flow cytometry analysis using ethanol-fixed, propidium iodide-stained cells. [B and D] Western blotting for expression of cyclin D1, cleaved caspase 3 or tubulin as a loading control was performed. [C] Induction of apoptosis was evaluated by staining of cells with propidium iodide and annexin V. [E] HO-1 silencing was evaluated with Western blotting. Controls represent expression of HO-1 in hemin-treated C-26 cells. HuHO-1 siRNA is a negative control targeting xenogeneic (human) HO-1 gene, muHO-1 siRNA is a murine sequence that knocks-down HO-1 expression in C-26 cells. [F and G] production of reactive oxygen species was evaluated with flow cytometry by measuring CM-H_2_DCFDA fluorescence in comparison with controls.

To get insight into the mechanism of cytostatic/cytotoxic effects induced by Zn(II)PPIX cell cycle analysis and induction of apoptosis were performed. Incubation of C-26 cells with Zn(II)PPIX for 48 or 72 h resulted in dose- and time-dependent reduction of cells in G1 phase of the cell cycle (Fig. 3A). This effect correlated with decreased cyclin D1 expression (Fig. 3B) and increased percentage of cells in sub-G1 phase (Fig. 3A). Propidium iodide and annexin V staining of C-26 cells incubated with 5 μM Zn(II)PPIX for 24–72 h indicated that the sub-G1 fraction of cells might represent apoptotic cells (Fig. 3C). The fraction of late apoptotic and necrotic cells increased from 10.9% in controls to 30.4% after 72 h incubation with Zn(II)PPIX. Western blotting analysis indicated that a 48-h incubation of C-26 cells leads to accumulation of cleaved (active) caspase-3 (Fig. 3D).

An influence of Zn(II)PPIX as well as knock-down of HO-1 expression with siRNA were used to get insight into the specificity of the Zn(II)PPIX-mediated effects. As shown in Fig. 3E siRNA against a murine HO-1 (moHO-1 siRNA) was effective in reducing the expression level of HO-1 in murine C-26 cells. A siRNA against a human gene (huHO-1 siRNA) was ineffective (Fig. 3E) as well as an irrelevant siRNA against enhanced green fluorescent protein (eGFP, not shown). Inhibition of HO-1 activity with Zn(II)PPIX resulted in increased ROS generation in C-26 cells (Fig. 3F). Similar effects were observed when HO-1 expression was knocked-down with a specific siRNA against HO-1 (Fig. 3G). The influence of Zn(II)PPIX on ROS formation was completely abrogated when C-26 were co-incubated with 50 μM bilirubin (Fig. 3F). Similarly, the influence of siRNA was significantly decreased by forced expression of HO-1 by pre-incubation of C-26 with 10 μM hemin (Fig. 3G).

### Zn(II)PPIX induces antitumor effects in a murine C-26 model

**Figure 4:**
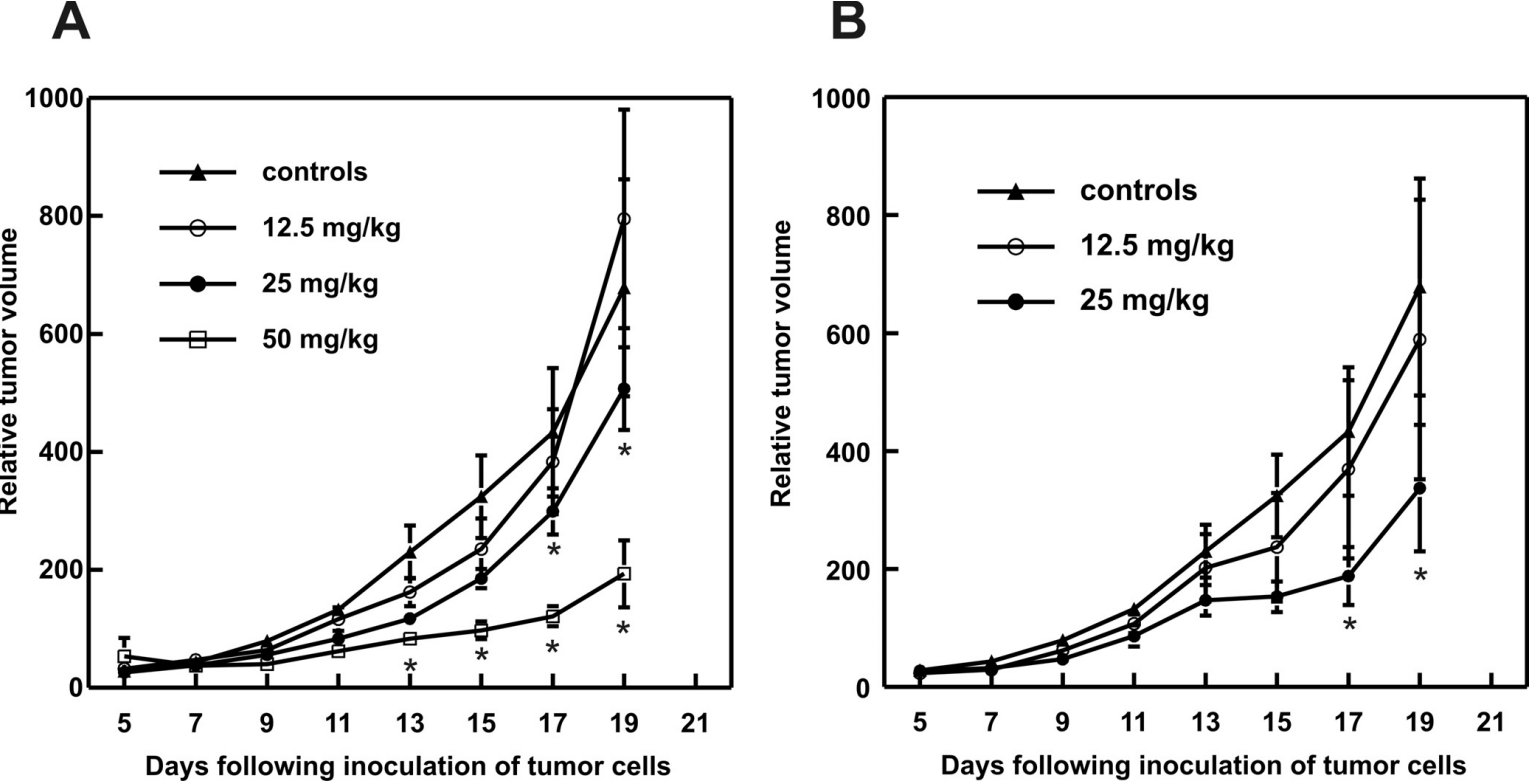
**Zn(II)PPIX exerts antitumor effects against C-26 adencarcinomas in mice**. BALB/c mice were inoculated with 1 × 10^5 ^of C-26 cells. On day 7 after inoculation of tumor cells mice were treated with Zn(II)PPIX administered i.p. or p.o. for 7 consecutive days [A and B]. Graphs show the influence of the treatment on the growth of C-26 tumors in mice. *P < 0.05 (two way Student's *t*-test) in comparison with controls.

BALB/c mice inoculated with C-26 cells were treated with Zn(II)PPIX for 7 consecutive days. HO-1 inhibitor was administered either intraperitoneally (i.p.) or *per os *and the tumor volume was monitored every second day, starting from day 7 after inoculation of tumor cells. Zn(II)PPIX exerted dose-dependent antitumor effects manifested by the retardation of tumor growth. A statistical significance was reached on days 17 and 19 for Zn(II)PPIX administered at a dose of 25 mg/kg either i.p. or orally (Fig. 4A and 4B). A stronger effect was observed when Zn(II)PPIX was administered i.p. at a dose of 50 mg/kg, where a statistically significant retardation of tumor growth was observed on days 13–19, as compared with controls (Fig. 4A).

### Overexpression of HO-1 in B16F10 cells confers resistance to cisplatin treatment

**Figure 5:**
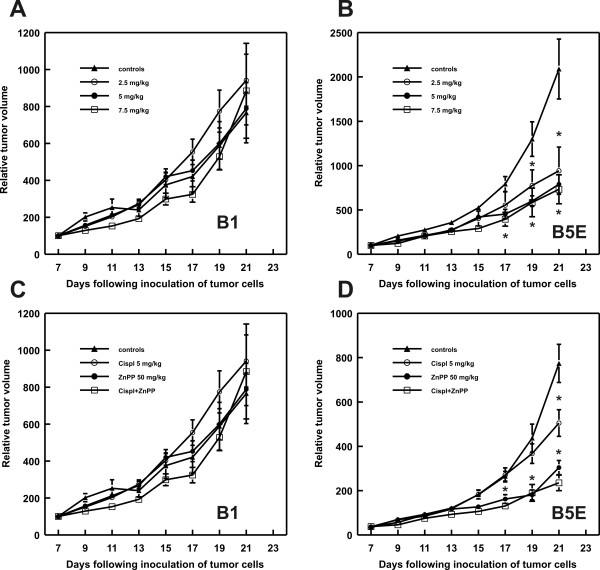
**Antitumor effect of cisplatin or the combined treatment with Zn(II)PPIX and/or cisplatin in mice transplanted with control or HO-1 overexpressing B16F10 tumors**. C57Bl/6 mice were inoculated with 1 × 10^6 ^of HO-1 overexpressing (B1) or mock-transfected (B5E) cells. On day 7 after inoculation of tumor cells mice were treated with cisplatin at a dose of 2.5, 5.0 or 7.5 mg/kg administered i.p. [A and B] or with cisplatin (5 mg/kg in a single i.p. injection) and 50 mg/kg of Zn(II)PPIX administered i.p. for 7 consecutive days [C and D]. Graphs show the influence of the treatment on the growth of melanoma in mice. *P < 0.05 (two way Student's *t*-test) in comparison with controls.

B16F10 cells were transfected with HO-1 gene (B1 clones) or an empty control plasmid (B5E cells). Tumor cells were inoculated with mixtures of clones and their response to cisplatin treatment was investigated. Cisplatin was administered i.p. at three doses of 2.5, 5.0 or 7.5 mg/kg and the tumor growth was monitored every second day. While in control B5E tumors cisplatin administration led to a significant retardation of tumor growth, the B1 tumors were completely resistant to the chemotherapeutic (Fig. 5A and 5B). Administration of 50 mg/kg Zn(II)PIX alone produced significant retardation of tumor growth only in B5E tumor model (Fig. 5D), but was completely ineffective in B1 tumors (Fig. 5C). Furthermore, it did not restore cisplatin sensitivity in B1 tumors (Fig. 5C).

### Zn(II)PPIX does not affect antitumor effects of chemotherapeutics

**Figure 6:**
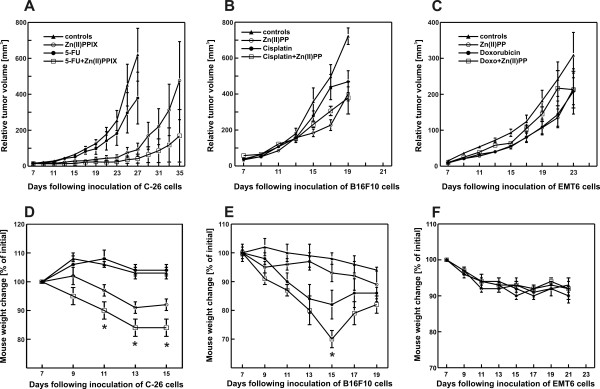
**Antitumor effect of the combined treatment with Zn(II)PPIX and/or chemotherapeutics**. Mice were inoculated with 1 × 10^5 ^of C-26 or EMT6 cells or with 1 × 10^6 ^of B16F10 cells. On day 7 after inoculation of tumor cells mice received a single dose of chemotherapeutics: 50 mg/kg of 5-FU [A, D], 5 mg/kg of cisplatin [B, E] or 7.5 mg/kg of doxorubicin [C, F]. Zn(II)PPIX was administered i.p. for 7 consecutive days at a dose of 50 mg/kg [A-F]. Graphs show the influence of the treatment on the growth of tumors in mice [A-C] or on the change in mice weight as compared with initial weight. *P < 0.05 (two way Student's *t*-test) in comparison all other groups.

In further studies the influence of Zn(II)PPIX (50 mg/kg) on the *in vivo *antitumor effects of chemotherapeutics was evaluated. Three different cell lines syngeneic with BALB/c or C57Bl/6 mice were used, namely C-26, B16F10 melanoma and EMT6 breast adenocarcinoma. The following chemotherapeutics were used in these studies: 5-fluorouracil at a dose of 50 mg/kg (5-FU, for C-26), cisplatin at a dose of 5 mg/kg (for B16F10) and doxorubicin at a dose of 7,5 mg/kg (for EMT6 cells). Although in *in vivo *studies administration of Zn(II)PPIX (at a dose of 50 mg/kg) resulted in retardation of tumor growth (although in EMT6 tumors the effect was only modest) there was no further potentiation of the antitumor effects by concomitant administration of Zn(II)PPIX together with chemotherapeutics (Fig. 6A–C). Only for Zn(II)PPIX and 5-FU a slightly stronger effect was observed for the combination treatment, but the difference between the combination and single drug-treated tumors did not reach statistical significance (Fig. 5A). Remarkably, the combined administration of Zn(II)PPIX with either cisplatin or 5-FU resulted in significant weight loss (Fig. 6D and 6E). This effects was not observed in mice treated with Zn(II)PPIX and doxorubicin (Fig. 6F). No treatment-related mortality was observed in these experiments.

## Discussion

The role of HO-1 in tumor development is still not completely elucidated and some recent reports demonstrate discordant or even completely opposite results. Nevertheless, accumulating evidence indicates that HO-1 is expressed or overexpressed by a large variety of human tumors and that it plays a critical role in progression of neoplastic diseases [7]. For example, it was shown that increased expression of HO-1 is associated with higher proliferation rate of various tumor cells [7,10,15,20], although opposite effects are observed in breast cancer cells [21]. Specific siRNA-mediated down-regulation of HO-1 resulted in suppression of proliferation of pancreatic cancer cells [10] or in induction of apoptosis of lung cancer cells [17]. Similarly, cytostatic/cytotoxic effects were observed with HO-1 inhibitors, such as Zn(II)PPIX [15,22] or its pegylated derivative (PEG-ZnPP) [16]. Moreover, overexpression of HO-1 increased viability, proliferation and angiogenic potential of melanomas [9]. Mice inoculated with HO-1 overexpressing melanomas fared worse than controls, had a higher number of metastases and a significantly shortened survival [9].

The results of present experiments performed with additional cell lines further establish that Zn(II)PPIX is an active agent capable of inducing cytostatic and cytotoxic effects against a number of human and mouse tumor cell lines. Moreover, in two different tumor models Zn(II)PPIX was demonstrated to exert potent antitumor effects manifested by the retardation of tumor growth.

Development of effective antitumor regimens requires administration of drugs in combination. The role of the combination treatment is to target different pathways in tumor cells in order to elicit more robust antitumor response. Synergistic antitumor effects might lead to decreased dosing and elimination or a significant attenuation of toxic side effects of drugs used in monotherapy. Several previous reports demonstrated that HO-1, which is frequently over-expressed in tumor cells, might participate in tumor-protective effects against a number of chemotherapeutics, radiotherapy and photodynamic therapy [10,11]. By targeting HO-1 it might be possible to sensitize tumor cells to more effective cytotoxic effects of other therapeutic regimens. Indeed, *in vitro *data seem to support this idea. HO-1 knock-down sensitized pancreatic cancer cells to gemcitabine [10] and lung cancer cells to cisplatin [17]. Moreover, PEG-ZnPP sensitized colon cancer cells to cytostatic/cytotoxic effects of camptothecin or doxorubicin [16]. However, the efficacy of combining chemotherapeutics with HO-1 inhibitor *in vivo *has not yet been addressed.

Considering the use of HO-1 inhibitors in combination with other antitumor agents it must be kept in mind that HO-1 and its products affect multiple signaling and metabolic pathways and play an important role in protection of normal cells against multiple environmental insults. For example, HO-1 protects retinal cells against light-induced damage [23], ameliorates ischemia-reperfusion injury elicited by a number of different conditions [24], inhibits endothelial cell apoptosis induced by endoplasmic reticulum stress [25], protects mice from apoptotic liver damage [26], prevents development of atherosclerosis in LDL-receptor knock-out mice [27], improves survival of transplanted organs [28] or prevents development of gastric ulcers in rats [29]. HO-1 also protects normal cells and tissues against toxic effects of chemotherapeutics. Heme-induced HO-1 expression protects against cyclophosphamide-mediated hemorrhagic cystitis [30], HO-1 attenuates cisplatin-induced toxicity in renal tubular cells [31] or in auditory cells [32], it also decreases doxorubicin-mediated cardiotoxicity [33]. Moreover, transgenic mice deficient in HO-1 (-/-), develop more severe renal failure and have significantly greater renal injury compared with wild-type (+/+) mice treated with cisplatin [34].

At doses used in the studies presented here Zn(II)PPIX was unable to restore cisplatin sensitivity in HO-1 overexpressing melanoma cells nor was it capable of potentiating antitumor effects of cisplatin, doxorubicin or 5-FU in three different models. Nonetheless, Zn(II)PPIX significantly potentiated toxicity of cisplatin and 5-FU as determined by decreased weight loss. Tozer *et al*, have shown that administration of Zn(II)PPIX at a dose of 45 μmoles/kg is ineffective in inhibiting HO-1 activity in tumors [35]. The dose used in these studies (50 mg/kg) corresponds to almost 80 μmoles/kg/mouse. Although it was not measured whether Zn(II)PPIX used at this dose and at this administration schedule was capable of inhibiting HO-1 activity it can be concluded that it is ineffective in neither inducing antitumor effects in HO-1-overexpressing B16F10 melanomas nor in restoring sensitivity to cisplatin. Future studies should address an important, and not addressed in these studies, question of drug administration schedule. Specifically, what should be the timing of Zn(II)PPIX administration in combination with chemotherapeutics to effectively inhibit HO-1 activity in tumor cells? Is it possible to design combination strategies that would target HO-1 in tumors and not in normal tissues? It seems that the influence of Zn(II)PPIX administration on the activity of HO-1 in tumor versus normal tissues will be of utmost importance in designing combination treatments.

## Conclusion

Altogether, these studies show that despite promising cytostatic/cytotoxic and even antitumor effects elicited by Zn(II)PPIX, this HO-1 inhibitor should not be used in combination with chemotherapeutics. It can be hypothesized however, that selective and efficient delivery of HO-1 inhibitors to the tumor might prove to be a more rational approach for combination therapies with chemotherapeutics. Indeed, as demonstrated by Fang *et al*. [16] and Iyer *at al*. [36] it is possible to prepare Zn(II)PIX derivatives with a higher tumor selectivity. It remains to be elucidated whether these strategies are more effective and less toxic.

## Competing interests

The authors declare that they have no competing interests.

## Authors' contributions

DN participated in experimental design, performed most of *in vitro *and *in vivo *experiments and participated in the writing of the manuscript, MB participated in *in vivo *experiments, MW and JB did most of the flow cytometry studies, AS measured ROS formation in tumor cells, PS participated in cytostatic/cytotoxic asssays *in vitro*, TI participated in *in vivo *experiments, HW prepared control and HO-1 overexpressing B16F10 melanoma cells, AJ participated in experimental design, JD participated in experimental design and participated in the writing of the manuscript, TS participated in experimental design and in *in vitro *experiments, and participated in manuscript correction, JG conceived the study, participated in its design and coordination and wrote the first draft of the manuscript. All authors read and approved the manuscript.

## Pre-publication history

The pre-publication history for this paper can be accessed here:


